# Steroid Nanocrystals Prepared Using the Nano Spray Dryer B-90

**DOI:** 10.3390/pharmaceutics5010107

**Published:** 2013-01-25

**Authors:** Koichi Baba, Kohji Nishida

**Affiliations:** Department of Ophthalmology, Osaka University Graduate School of Medicine, 2-2 Yamadaoka, Suita, Osaka 565-0871, Japan; E-Mail: knishida@ophthal.med.osaka-u.ac.jp

**Keywords:** steroid, fluorometholone, dexamethasone, drug nanocrystals, Nano Spray Dryer B-90

## Abstract

The Nano Spray Dryer B-90 offers a new, simple, and alternative approach for the production of drug nanocrystals. In this study, the preparation of steroid nanocrystals using the Nano Spray Dryer B-90 was demonstrated. The particle size was controlled by selecting the mesh aperture size. Submicrometer steroid particles in powder form were successfully obtained. These nanoparticles were confirmed to have a crystal structure using powder X-ray diffraction pattern analysis. Since drug nanocrystals have recently been considered as a novel type of drug formulation for drug delivery systems, this study will be useful for nano-medical applications.

## 1. Introduction

The use of drug nanoparticles as a drug delivery system has attracted considerable attention in the field of nanomedicine. Nanoparticles in which the drug filling rate is 100% (where the amorphous form is also filled) are called drug nanocrystals [1]. Since nanocrystals have a large surface area compared with microparticles, drug nanocrystals have several unique properties, including increased dissolution velocity, increased saturation velocity, and increased adhesion to cell membranes [2]. Additionally, drug nanocrystals enable larger amounts of drugs to be delivered into cells and tissues at a single-particle level, because of their densely packed crystal structure [2]. Because of their unique physicochemical properties, drug nanocrystals have recently been considered as a novel type of drug formulation for drug delivery systems [3].

Generally, since the spray dryer technique uses a facile approach for the preparation of nanoparticles—involving spraying, the evaporation of the ethanol-based drug solution, and the collection of the drug particles—numerous drugs are feasible candidates for the preparation of nanoparticles using this technique. However, it is difficult to prepare particles smaller than 2 µm using conventional spray dryer techniques, and it is also difficult to collect the finer particles [4]. In other words, submicrometer-sized particles, *i.e.*, nanoparticles, cannot be produced using conventional spray dryers. Recently, an advanced spray dryer technology, the Nano Spray Dryer B-90, was developed by Büchi^®^ [5]. The piezoelectrically driven vibrating mesh and the electrostatic particle collector allow the successful preparation and collection of nanoparticles. The different mesh aperture sizes can be used to create different sizes of nanoparticles. To date, drug-encapsulated polymeric nanoparticles [6], protein nanoparticles [7], and lithium carbonate (Li_2_CO_3_) hollow spheres used in lithium batteries [8] have been successfully prepared using the Nano Spray Dryer B-90. 

Recently, our interest has focused on the development of a novel type of steroid nanocrystal-based eye drops, used to treat ophthalmic diseases. A number of steroid compounds are hydrophobic by nature, including fluorometholone and dexamethasone. They are therefore used in ophthalmic treatments in the form of an eye drop suspension formulation of large particles (*i.e*., more than several micrometers in size). These commercially available eye drops certainly show drug efficacy against the inflammation of eye diseases. However, the ocular penetration of these steroid drugs is considered to be comparably low, because of the low dissolution velocity of the drug particles, which results from their large size (approximately 6 µm) [9]. These micron-sized particles are produced via a milling process, owing to industrial compromises made to achieve reductions in costs. It is reported that if the drug particle size is reduced to less than 2 µm, the total dissolution velocity of the drugs will be increased, resulting in increases in the drugs’ ocular penetration [9]. The high ocular penetration of drugs is useful in achieving high drug efficacy, as well as in reducing side effects by allowing doses to be minimized. The preparation of steroid particles smaller than ~2 µm is therefore an attractive approach for producing effective drug formulations with high drug efficacy. The production of nanocrystals, which have a size defined as between a few nanometers and 1000 nm (= 1 μm) [1], is especially attractive for these purposes. 

**Figure 1:**
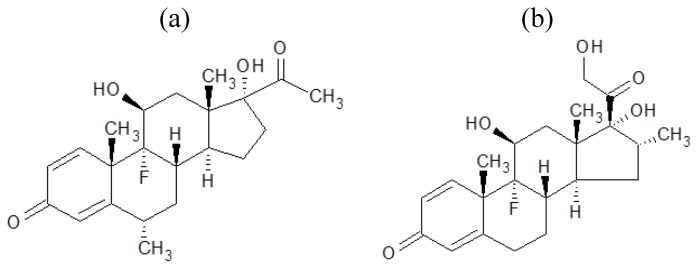
Chemical structures of (**a**) fluorometholone and (**b**) dexamethasone.

In this research, we demonstrated the preparation of steroid nanocrystals in powder form, using the Nano Spray Dryer B-90. The steroid drugs selected were fluorometholone (Figure 1a) and dexamethasone (Figure 1b). Since it is known that the mesh aperture size is important in determining the resulting particle size [10], we investigated the relationship between mesh aperture size and drug particle size. To confirm that the nanocrystals did indeed have a crystal structure, powder X-ray diffraction pattern analysis was carried out.

## 2. Experimental Section

### 2.1. Materials and Methods

The fluorometholone, dexamethasone, and ethanol (99.5%, *v*/*v*) were purchased from Wako Pure Chemical Industries (Osaka, Japan). The fluorometholone and dexamethasone were dissolved in ethanol at concentrations of 1 mg/mL with a final volume of 10 mL and 10 mg/mL with a final volume of 12.5 mL, respectively. Since there was the fact that the fluorometholone did not soluble to the ethanol as much as that of the dexamethasone, the concentration of the ethanol solution of fluorometholone was tuned to be lower than that of dexamethasone. 

### 2.2. Preparation of Nanocrystals Using the Spray Dryer B-90

**Figure 2:**
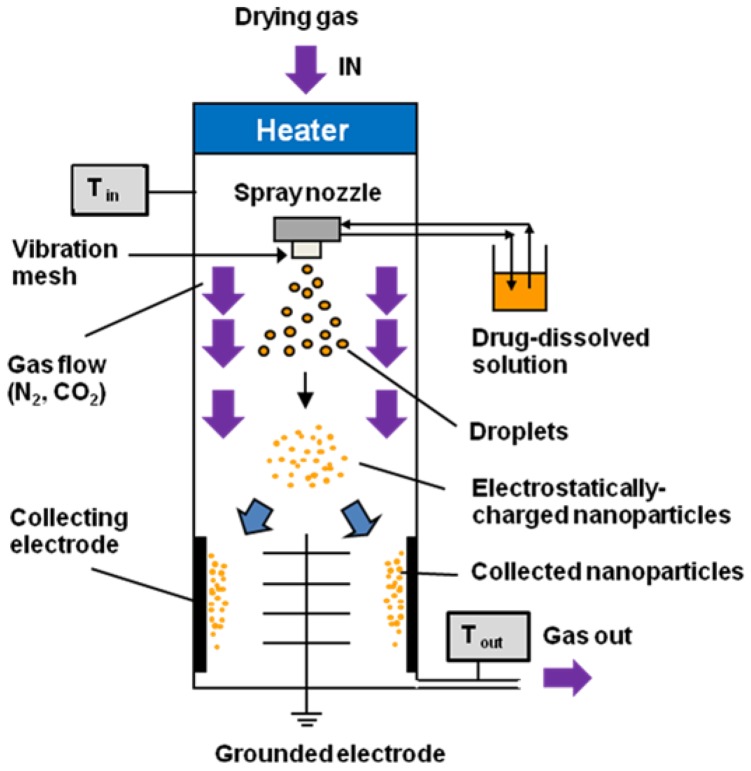
Schematic image of the Nano Spray Dryer B-90.

The ethanol-dissolved drug solutions were then used to prepare the nanocrystals, using the Nano Spray Dryer B-90 (Büchi^®^). A schematic image of the Nano Spray Dryer B-90 is shown in Figure 2. Briefly, the drying gas, which is heated up to the setting inlet temperature, flows into a drying chamber. The gas then exits from the spray dryer, passing through the clearing filter at the bottom. The inlet temperature and outlet temperature are shown as T_in_ and T_out_, respectively. The operating conditions for the experiments were kept constant at T_in_ = 50 °C, T_out_ = 35 °C, feed rate 25 mL/h, drying gas flow rate = 100 L/min. Spray mesh aperture sizes of 4.0, 5.5, and 7.0 μm were used in these experiments. Finally, the resulting nanocrystal powders were collected using a rubber spatula. 

### 2.3. Scanning Electron Microscopy Observation of the Nanocrystals

The morphology and size of the collected particles were observed using scanning electron microscopy (SEM; JEOL-6510LA). The average size and particle size distribution were calculated by counting more than 300 particles from the obtained SEM pictures.

### 2.4. Powder X-ray Diffractometry Analysis

The crystal structure of the nanocrystals was confirmed using powder X-ray diffractometry (SmartLab, Rigaku). CuKα radiation (1.54 Å) was used as an X-ray source. The X-ray output was 45 kV and 200 mA. Statistical analysis (a two-tailed t-test) was carried out to clarify the difference between the diameter of the nanoparticles and the aperture size of the apparatus (Figure 5 and Figure 6). 

## 3. Results and Discussion

The ethanol-dissolved drug solution was fed to the spray head by pumping. The solution was atomized by piezoelectrically driven mesh vibrations in the small spray cap, and millions of precisely sized droplets (with a narrow size distribution) were ejected each second by the vibrating actuator, which was driven at approximately 60 kHz. The extremely fine droplets dried to form solid particles during their passage through the chamber; these particles were then electrostatically charged by dry N_2_ and CO_2_ gases, and were collected by the electrode. A schematic image of the Nano Spray Dryer B-90 is shown in Figure 2. 

**Figure 3:**
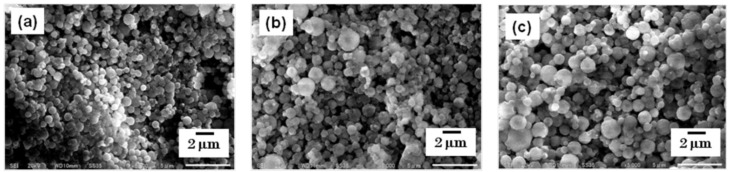
SEM images of fluorometholone nanocrystals. The nanocrystals were prepared using mesh aperture sizes of (**a**) 4.0 µm, (**b**) 5.5 µm, and (**c**)7.0 µm.

**Figure 4:**
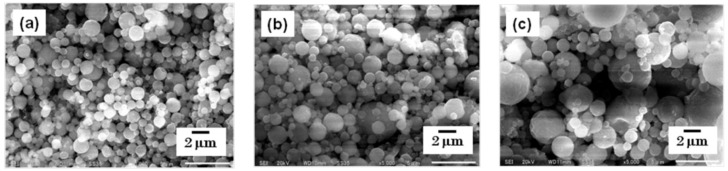
SEM images of dexamethasone nanocrystals. The nanocrystals were prepared using mesh aperture sizes of (**a**) 4.0 µm, (**b**) 5.5 µm, and (**c**) 7.0 µm.

The particles were collected using a rubber spatula, and were observed using SEM. The morphology of each steroid nanocrystal was sphere-like, regardless of the mesh aperture size, which was varied between 4.0, 5.5, and 7.0 µm (Figure 3 and Figure 4). 

**Figure 5:**
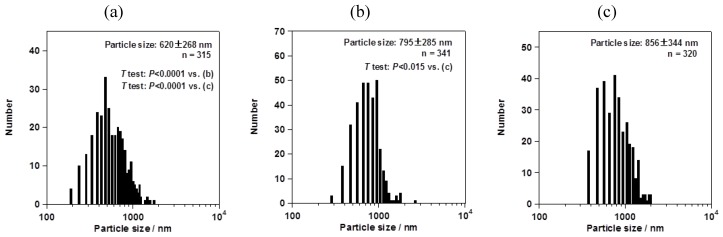
Average sizes of fluorometholone nanocrystals, with their size distributions. The mesh aperture sizes used were 4.0 µm (**a**), 5.5 µm (**b**), and 7.0 µm (**c**). The “*n* =” inserted in the figure represents the number of counted particles. The error bars associated with the particle sizes show the standard deviation.

**Figure 6:**
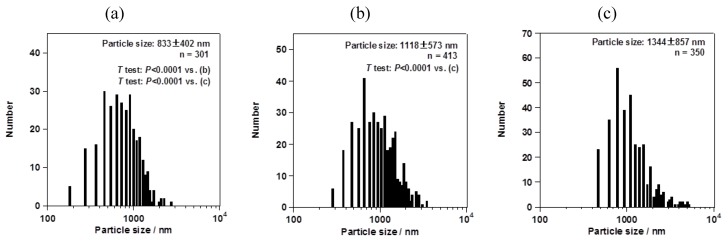
Average sizes of dexamethasone nanocrystals, with their size distributions. The mesh aperture sizes used were (**a**) 4.0 µm, (**b**) 5.5 µm, and (**c**) 7.0 µm. The “*n* =” inserted in the figure represents the number of counted particles. The error bars associated with the particle sizes show the standard deviation.

However, the particle size changed depending on the mesh aperture size. For the fluorometholone nanocrystals, the average particle sizes with their size distribution were 620 ± 268, 795 ± 285, and 856 ± 344 nm for mesh aperture sizes of 4.0, 5.5, and 7.0 µm, respectively (Figure 5). For the dexamethasone nanocrystals, the average particle sizes with their size distribution were 833 ± 402, 1118 ± 573, and 1344 ± 857 nm for mesh aperture sizes of 4.0, 5.5, and 7.0 µm, respectively (Figure 6). For both the fluorometholone and dexamethasone particles, the size distribution of the particles became narrower with decreasing mesh aperture size. The size and size distribution for each sample was significantly different from those of each other sample (see *p* values in Figure 5 and Figure 6). The validity of these results are supported by previous reports, which showed that different mesh aperture sizes resulted in different sizes of particles; the average particle size decreased with decreasing mesh aperture size [10]. This is because smaller mesh aperture sizes tend to generate smaller droplets of ethanol-dissolved drug solution, compared with large mesh apertures. Figure 5 and Figure 6 suggest that the increased size of the dexamethasone particles (compared with the fluorometholone particles) might have resulted from the difference in the concentrations of the ethanol-dissolved drug solutions. The concentrations of the ethanol solutions of fluorometholone and dexamethasone were 1 mg/mL and 10 mg/mL, respectively; *i.e.*, the concentration of the dexamethasone solution was 10 times higher than that of the fluorometholone solution. The obtained results seem reasonable when compared with previous reports [10,11], in terms of the fact that the particle size was significantly affected by the concentration of the drug solutions. The particle size tended to increase with increasing drug concentration. The details of the effect of the drug concentration on the resulting steroid compound particle sizes will be discussed elsewhere. 

**Figure 7:**
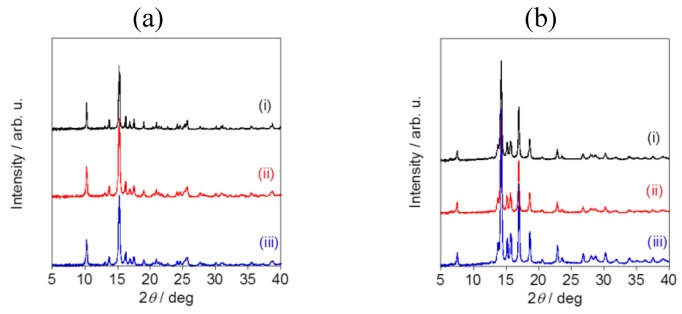
Powder X-ray diffraction pattern analysis of (**a**) fluorometholone and (**b**)dexamethasone nanocrystals. The mesh aperture sizes used were (**i**) 4.0 µm, (**ii**) 5.5 µm, and (**iii**) 7.0 µm.

Powder X-ray diffraction pattern analysis revealed the specific diffraction patterns for each sample, confirming that all of the fluorometholone and dexamethasone nanocrystals had a crystal structure (Figure 7). Although the particle sizes were different, the crystal structures were same for the fluorometholone and dexamethasone nanocrystal samples (Figure 7a,b). We successfully prepared size-controlled fluorometholone and dexamethasone nanocrystals using the Nano Spray Dryer B-90, by selecting the mesh aperture size. 

## 4. Conclusions

We succeeded in preparing size-controlled steroid nanocrystals using the Nano Spray Dryer B-90. The particle size was controlled by the mesh aperture size; when the mesh aperture size was decreased, the particle size decreased. Powder X-ray diffraction pattern analysis confirmed that the nanocrystals had a crystal structure, which showed specific diffraction patterns. The detailed experimental conditions, including the concentration of the ethanol-dissolved drug solution, the inlet temperature, and the drying gas flow rate, that might affect the particle formation will be investigated in future work, as will the dissolution velocity of the drug nanocrystals in aqueous media. Additionally, we will investigate the possibility of the polymorphs and phase transitions of nanocrystals by differential scanning calorimetry (DSC) measurement. This DSC data will provide information about the relationship between polymorphs/phase transition and dissolution profiles of drug nanocrystals. We are also investigating the preparation of aqueous dispersions of steroid nanocrystals. In this stage, the detailed particle size distribution in aqueous medium will be analyzed by dynamic light scattering measurement. These aqueous nanocrystal dispersions will be attractive as nanocrystal-based eye drop solutions with the potential to be used for the treatment of ophthalmic disorders in the near future. We expect the drug nanocrystals to be used in drug delivery systems as nano-medical applications.
